# High-dose intravenously administered iron versus orally administered iron in blood donors with iron deficiency: study protocol for a randomised, controlled trial

**DOI:** 10.1186/s13063-016-1648-y

**Published:** 2016-10-28

**Authors:** Susanne Macher, Camilla Drexler, Ines Lindenau, Nazanin Sareban, Peter Schlenke, Karin Amrein

**Affiliations:** 1 Department of Blood Group Serology and Transfusion Medicine, Medical University of Graz, Graz, Austria; 2 Department of Internal Medicine, Division of Endocrinology and Diabetology, Medical University of Graz, Auenbruggerplatz 15, 8036 Graz, Austria

**Keywords:** Blood donation, Donor safety, Intravenous iron, Iron carboxymaltose, Iron deficiency, Randomised controlled trial

## Abstract

**Background:**

About 2–3 % of the population participates in blood donation programmes. Each whole blood donation or ten apheresis donations cause a loss of 200–250 mg of iron. As a result, one of the most common risks of regular blood donors is iron deficiency. Although this has been known for decades, in most countries, iron status is currently not assessed or treated in this population. Premenopausal women are particularly affected, as they have lower iron reserves and higher daily requirements. Besides anaemia, iron deficiency may lead to fatigue and impaired cognitive and physical performance. Current iron preparations for intravenous administration are well tolerated and allow for application of large doses up to 1 g in one visit. Our hypothesis is that in blood donors with iron deficiency, intravenously administered iron is more efficient and as safe as oral iron supplementation. Since anaemia is one of the most frequent reasons for permanent or intermittent donor deferral, maintaining an iron-replete donor pool may help to prevent shortages in blood supply and to avoid iron deficiency-related comorbidities.

**Methods/design:**

In this randomised clinical trial we include male and female blood donors aged ≥18 and ≤65 years with a ferritin value of ≤30 ng/ml. Stratified by gender, participants are randomized with a web-based randomisation tool in a 1:1 ratio to either 1 g of intravenously administered ferric carboxymaltose or 10 g of iron fumarate supplements at one to two daily doses of 100 mg each. Eight to 12 weeks after the first visit, iron status, blood count and symptoms are assessed in both groups. The primary endpoint is the difference in transferrin saturation (%) following the intervention between both groups. Secondary endpoints include other parameters of iron metabolism and red blood cell count, the number of patients with drug-related adverse events, and subjective symptoms including those of the restless legs syndrome, quality of life, and fatigue.

**Discussion:**

Iron supplementation administered intravenously in non-anaemic but iron-deficient blood donors could represent an effective strategy to protect blood donors from comorbidities related with iron deficiency and therefore improve blood donor wellbeing. Furthermore, iron supplementation will help to maintain an iron-replete blood donor pool.

**Trial registration:**

EudraCT: 2013-000327-14, Clinical Trials Identifier: NCT01787526. Registered on 6 February 2013.

**Electronic supplementary material:**

The online version of this article (doi:10.1186/s13063-016-1648-y) contains supplementary material, which is available to authorized users.

## Background

Iron deficiency is possibly the most prevalent nutritional deficiency worldwide, but guidelines for its diagnosis and treatment vary in their suggestions [1, 2]. The largest part of iron is bound to haemoglobin in red blood cells [1]. Each whole blood donation (plus additional samples for testing) causes an iron loss of 200 to 300 mg [3]. Therefore, iron deficiency is prevalent among healthy blood donors, who represent up to 2–3 % of the population [4–10]. Contributing risk factors for iron deficiency include donation frequency, low body weight, and female gender [6, 8, 9]. Traditionally, safety issues in transfusion medicine have been concentrating on product quality and patient safety. However, there is still room for optimizing donor care [11]. In Austria, the maximal annual donation frequency for whole blood donations is 4 times for premenopausal women, 5 times for postmenopausal women and 6 times for men. The maximal annual donation frequency for platelet apheresis is 26 times and for plasmapheresis 50 times [12]. Recently, it has been shown that without iron supplementation, whole blood donors do not recover their predonation hemoglobin and iron status within 6 months [13]. Iron depletion in blood donors has been recognized for decades [14], and anaemia is routinely assessed by point-of-care haemoglobin devices, but iron status is not. Therefore, non-anaemic iron-deficient donors may not be identified, as iron deficiency may lead to subtle and unspecific symptoms [15].

Several trials have evaluated different regimens of iron supplementation in blood donors and demonstrated good treatment efficacy in improving iron status [13, 16–24]. However, many iron-deficient blood donors may need to permanently take oral iron if they continue to donate frequently, since it typically takes 2–3 months to correct iron deficiency, which is the minimal time interval between whole blood donations. High-dose iron preparations for intravenous administration have become available which allow for the application of a large dose of 1000 mg in one visit, correcting iron deficiency in many individuals with a single infusion [25]. To our knowledge, only two interventional trials using high-dose iron intravenous administration have been performed in blood donors, but only in one study was iron deficiency required for inclusion [26, 27]. Currently, intravenous iron is not routinely accessible to iron-deficient blood donors, and there are no studies that have compared intravenously administered iron to oral iron supplements in this setting. In our study, we aim to establish that intravenous iron is feasible for correction of iatrogenic iron deficiency in blood donors. When costs and logistical impediments are overcome, this could be a one-stop approach if intravenous iron is given immediately after donation through the available venous access.

The potential risks related to iron therapy include the masking of other causes of iron deficiency, iron overload in unrecognized hemochromatosis, and drug-related adverse effects. Results from interventional trials in other settings indicate that severe drug reactions of oral and intravenous iron are rare, and that gastrointestinal side effects are experienced less often with intravenously administered iron than with oral iron supplements [25, 28]. However, anaphylactic reactions have been described in the intravenous administration of iron [29]. A novel, frequent adverse effect after the intravenous infusion of iron and more frequently after ferric carboxymaltose infusion is hypophosphatemia, which may be severe and can lead to profound weakness and even osteomalacia [30–33].

### Aims and hypothesis of the study

This study will assess the efficacy, safety, and feasibility of intravenous infusion of high-dose iron in blood donors with iron deficiency by comparing intravenously administered ferric carboxymaltose with oral iron fumarate supplements.

Our hypothesis is that 1 g of intravenously infused ferric carboxymaltose will improve the iron status of blood donors with iron deficiency more efficiently compared to a comparable dose of oral iron supplements (10 g assuming a 10 % resorption rate, given over 8–12 weeks).

## Methods/design

A SPIRIT checklist is available online for this manuscript (Additional file 1).

### Study design

This is a prospective, randomised, clinical trial. The study population consists of healthy adults, including male and female whole blood and platelet apheresis donors with iron deficiency (ferritin ≤ 30 ng/ml).

### Participants

#### Inclusion criteria

The inclusion criteria are as follows:

- Age ≥ 18 years and ≤ 65 years
- Iron deficiency (ferritin ≤30 ng/ml at screening — usually before blood donation)
- Fulfilment of the Austrian criteria for blood donation

#### Exclusion criteria

The exclusion criteria are as follows:

- Known haemochromatosis
- Acute infection
- Pregnancy or lactation
- History of anaphylaxis to intravenous infusion of iron or other substances
- Signs or symptoms suggestive of acute or chronic gastrointestinal or excessive gynaecological bleeding

### Study drugs/intervention

The study drugs used in the intervention are:

- **High-dose (1000 mg) intravenous infusions of iron** (ferric carboxymaltose, Ferinject ®, manufacturer Vifor Pharma, Austria) or
- **Oral supplements of iron** (iron fumarate, Ferretab®, manufacturer Gerot Lannach, Austria) in a corresponding estimated dose of >10.000 mg (assuming an absorption of 10 %, 100 capsules of 152 mg iron fumarate each) taken over 8–12 weeks. Participants are advised to take tablets in the morning with orange or lemon juice.

### Outcomes

#### Primary endpoint

- The primary endpoint is the difference in transferrin saturation (%) at visit 1 between the high-dose intravenous infusion and the oral iron supplement group

#### Secondary objectives

The secondary objectives are:

- Other parameters of iron metabolism, red blood cell count including the percentage of participants with a normal (gender-specific) haemoglobin at visit 1 (group comparison) and further parameters (e.g. phosphate)
- Symptoms including those of restless legs syndrome, quality of life, fatigue
- Comparison of the two treatments regarding the incidence and severity of adverse events, tolerance, and compliance

### Methods of procedure

**Fig. 1:**
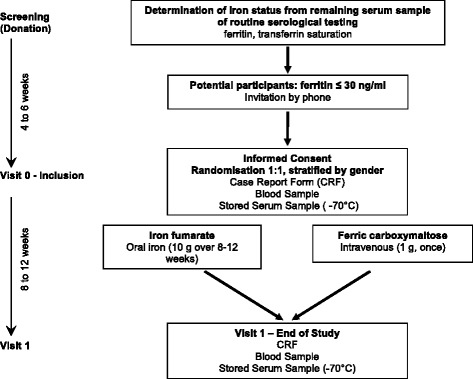
Study flow chart

A flow chart of the study procedure is given in Fig. 1. The total study duration for each patient will be 8–12 weeks. Based on previous experiences in the recruitment of blood donors for observational studies, we aim to include two study participants per week. Thus, the total recruitment period is estimated to last for 21–24 months and the total active study duration will be 26 months. Recruitment started in June 2014 and was completed in June 2016. At the time of revision, all 176 participants (172 according to the sample size calculation plus 4 dropouts) have been included.

### Study duration for the individual participant

The active treatment period for each participant is 8–12 weeks.

### Screening

Typically after a regular whole blood or platelet apheresis donation, ferritin is measured from the remaining serum for routine infectious disease testing. The regular written informed consent for blood donation covers the additional ferritin analysis. Donors with a serum ferritin ≤30 ng/ml will then be invited by telephone to participate in the study.

### Visit 0 including randomization procedure (4–6 weeks after blood donation)

Donors willing to participate are requested to visit the Apheresis Unit of the Department of Blood Group Serology and Transfusion Medicine. After giving written informed consent, checking inclusion/exclusion criteria and performing a urine pregnancy test in premenopausal females, a case report form (CRF) is filled out by the donor.

The CRF includes questions on the following aspects:

- History of blood donation (total number, time)
- History of anaemia
- Symptoms of fatigue
- Subjective physical and cognitive performance including self-reported physical activity and hours worked per week
- Symptoms of depression or lethargy
- Trophic changes of fingernails or hair
- Fatigue induced by blood donation
- Symptoms of restless legs syndrome

#### Randomization procedure

Each participant will be randomized in a 1:1 ratio (stratified by gender) to either high-dose parenterally infused iron or oral iron supplements using a web-based randomisation tool (http://www.randomizer.at). Good clinical practice (GCP) compliance of this software was confirmed by the Austrian Agency for Health and Food Safety (AGES).

#### Blood samples

A blood sample (~30 ml) is drawn from all participants, in case of randomisation to parenterally infused iron from a venous access placed for the application of the intravenously administered iron. Some parameters may be measured after the end of the study from frozen blood specimens stored at −70 °C.

#### Faecal occult blood tests

To assess occult gastrointestinal bleeding, all participants are asked to perform three faecal occult blood tests at home in the time between visit 0 and visit 1.

#### Medication

According to the randomization, either 100 tablets of oral iron supplements are handed out to the participant or iron is administered intravenously by the study investigators in the presence of a nurse.

For oral iron supplements, all participants receive an exact schedule for tablet intake together with clear instructions according to the package leaflet: we suggest to take 10 tablets per week (3 days 2 tablets, 4 days 1 tablet) and to take the tablets with orange juice.

Iron is administered intravenously to the participants in a bed in a separate room of the Apheresis Unit of the University Clinic of Blood Group Serology and Transfusion Medicine. Iron is infused intravenously over 30 minutes or more over a dedicated peripheral line. A standard operating procedure involving the resuscitation outreach team of the Department of Anesthesiology has been developed, and basic emergency equipment is available at all times.

### Visit 1 (8–12 weeks after visit 0)

A treatment period of 8–12 weeks is chosen because the administration of 8–13 doses of 100 mg iron in one week is feasible and it also matches the typical donation interval. Furthermore, serum ferritin levels are falsely high when analysed ≤6 weeks after intravenous infusion of iron.

A CRF including assessment of compliance is completed by the donor, and a blood sample (~30 ml) is drawn. All results are summarized in a written report for the study participants and sent by regular mail.

The CRF at visit 1 will additionally include questions on the following aspects:

- Information on any relevant adverse events, serious adverse events and adverse drug reactions
- Compliance with oral study medication intake (self-reported; the participants are also asked to bring back all leftover medication)

### Statistical aspects

#### Sample size justification

The sample size calculation was performed by the Department of Medical Informatics, Statistics and Documentation of the Medical University of Graz. Using a two-group *t* test with a 0.05 two-sided significance level, a sample size of 86 participants in each group will have 90 % power to detect a difference in mean of 8 % in transferrin saturation (estimating a post-treatment transferrin saturation of 28 % and 20 % in the high-dose intravenous iron group and oral iron group, respectively, based on results by a similar treatment regimen in otherwise healthy women with postpartum anaemia [34]). Allowing for a dropout of 14 %, originally 200 participants were planned to be enrolled in the study. As the actual dropout rate was substantially lower (<3 %), the study will be terminated after complete study participation of 172 participants plus the number of dropouts.

#### Statistical procedures

Statistical analyses of the results will be performed using the intention-to-treat and the per-protocol principles. All study participants who are randomised and included into the study will be analysed whether they take their study medication for the entire term (oral iron) or not or whether or not they miss the follow-up visit (visit 1). All primary and secondary outcome measures will be analysed and checked for normal distribution of the results. If there is a normal distribution of laboratory results, they will be analysed by the two-sided *t* test; if there is not a normal distribution they will be analysed by non-parametric tests like the Wilcoxon test for related samples or the Mann-Whitney U test for comparison of independent samples. The significance level will be set to *p* < 0.05. Data will be presented as mean ± standard deviation or median (interquartile ranges) as appropriate. For missing data, no imputation will be performed. A statistical evaluation will be performed using the software program SPSS for Windows (IBM SPSS Inc., Chicago, IL, USA).

A predefined subgroup analysis will separately analyse participants with a baseline ferritin of ≤15 ng/ml. The Department of Blood Group Serology and Transfusion Medicine, Medical University of Graz, will maintain responsibility for the final trial dataset.

### Data and safety monitoring

All laboratory analyses are stored on password-protected secure servers and will only be accessible for authorized individuals from the Medical University of Graz. Clinical data are collected through paper-based CRFs. The CRFs are confidential documents and held securely at the Department of Blood Group Serology and Transfusion Medicine. Each participant is assigned a trial identity code number to anonymize documented CRF data.

In the CRF of the follow-up visit (visit 1), the participants are asked for possible drug-related adverse events. Furthermore, all participants are requested to promptly report possible adverse events by telephone. The study participants receive telephone contact numbers from the study team at the time of inclusion (visit 0).

### Subject recruitment

In Styria, annually about 60.000 units of whole blood are collected at mobile and stationary locations by the Red Cross and further processed to packed red blood cell concentrates, pooled platelet concentrates and fresh frozen plasma at the Department of Blood Group Serology and Transfusion Medicine of the Medical University of Graz. Approximately 1200 single donor platelet apheresis collections are performed in our department per year. A number of different commercial plasma collection centres in the region offer the opportunity to donate apheresis plasma.

Depending on the region of residence, we estimate that 5000 to 10,000 donors are potential participants of this study. Of those, 1000 to 2000 may fulfil the inclusion and exclusion criteria; thus, we need to recruit 10–20 % of all potential participants. We aim to recruit two blood donors per week for this trial.

The University Hospital of Graz (“LKH-Universitätsklinikum Graz”) is a large tertiary care facility with 7200 employees. It is the teaching hospital for one of the three public Medical Universities in Austria. The catchment area covers the entire southeast of Austria (approximately 2 million people). Currently 1413 beds at general wards and 112 intensive care unit (ICU) beds are available. Each year, approximately 80,000 patients are treated as inpatients and 440,000 as outpatients.

### Specific gender relevance of the project

Adverse reactions related to blood donation are more common in females, which is one of the major reasons why more men become regular donors [35]. In particular, iron deficiency caused by blood donation is highly prevalent in premenopausal female donors because of lower blood volume, higher requirements, and greater losses during menstrual bleeding, pregnancies, birth, and lactation [6, 8, 9, 36, 37]. The estimated iron loss from one whole blood donation corresponds to approximately 10 % of total body iron and 66–97 % of the total iron stores of a premenopausal woman [38, 39].

More importantly, unrecognized and therefore untreated iron deficiency has been linked to adverse pregnancy outcomes such as preterm delivery and lower weight babies [40]. Particularly in the subgroup of premenopausal females, a regular evaluation of donor iron status should be considered or the donation intervals prolonged.

#### Study participation for premenopausal women

There are no data available on foetal toxicity of ferric carboxymaltose, so the application of this drug during the early stages of pregnancy is contraindicated. Therefore, women of childbearing age are only allowed to participate in this trial when pregnancy has been excluded by urinary β-HCG testing at the beginning of the study and is repeated by the participants in the intervention phase. Lactating women are excluded from blood donation and therefore are also not targeted in this study.

### Ethical aspects

The approval of the institutional ethical committee was given in June 2013 (EK 25–345 ex 12/13) and has been renewed yearly.

### Registration at clinical trials

This trial was registered at ClinicalTrials.gov in February 2013 (Clinical Trials Identifier: NCT01787526).

## Discussion

Iron deficiency anaemia occurs when iron stores have been consumed [1]. Before establishment of anaemia, iron deficiency may have other, more subtle and unspecific adverse effects on an individual such as decreased physical and cognitive performance [41, 42]. Since iron is an important element in many enzymes, deficiency may affect various cellular processes like DNA synthesis [43], mitochondrial energy metabolism [44], and the immune system [45]. Therefore, blood donation establishments should focus on donors at risk for iatrogenic iron deficiency without anaemia and develop strategies for its prevention. Although the benefit of iron therapy in subjects without anaemia remains controversial, mainly due to a lack of large randomised clinical trials for truly deficient subjects, a few studies have reported interesting results. For example, iron supplementation reduced unexplained fatigue in otherwise healthy women with iron deficiency and improved verbal learning and memory in adolescent girls [15, 46]. Iron deficiency has additionally been found to be associated with restless legs syndrome, and iron therapy improved symptoms significantly in blood donors [47]. Recently, it was shown that the recovery of blood donors’ iron stores receiving oral iron supplementation after blood donation took a median of 76 days compared to a median recovery time of longer than 168 days for study participants not taking iron [13], also questioning the current relatively short donation intervals of 2–3 months.

So far, no randomised controlled trial has compared high-dose intravenous iron to oral iron supplementation in blood donors with iron deficiency. Intravenous iron may be an excellent option in this setting. If our study can establish that intravenous iron is feasible in blood donors and the substantial impediments like costs and logistics can be overcome, high-dose intravenously infused iron given immediately after whole blood or apheresis donation through the present venous access could be a very efficient way for correction and/or prevention of iatrogenic iron deficiency. Such an approach could protect blood donors from symptoms related to iron deficiency and help to maintain an iron-replete blood donor pool.

### Trial status at the time of initial manuscript submission

Recruitment for this trial is ongoing.

## Additional file

Additional file 1: SPIRIT 2013 Checklist: Recommended items to address in a clinical trial protocol and related documents*. (DOCX 63 kb)

## Abbreviations

AGES: Austrian Agency for Health and Food Safety
CRF: Case report form
GCP: Good clinical practice
ICU: Intensive care unit
IV: Intravenous
PO: Peroral
β-HCG: Beta-human chorionic gonadotropin
